# A clinical staging model for bipolar disorder: longitudinal approach

**DOI:** 10.1038/s41398-020-0718-9

**Published:** 2020-01-29

**Authors:** Lorena de la Fuente-Tomás, Pilar Sierra, Mónica Sanchez-Autet, Belén Arranz, Ana García-Blanco, Gemma Safont, Maria P. García-Portilla

**Affiliations:** 1 Centro de Investigación Biomédica en Red de Salud Mental (CIBERSAM), Madrid, Spain; 2 Department of Psychiatry, University of Oviedo, Oviedo, Spain; 3 Instituto de Investigación Sanitaria del Principado de Asturias (ISPA), Oviedo, Spain; 4 Instituto de Neurociencias del Principado de Asturias (INEUROPA), Oviedo, Spain; 5 La Fe University and Polytechnic Hospital and University of Valencia, Valencia, Spain; 6 Parc Sanitari Sant Joan de Deu, Barcelona, Spain; 7 University Hospital Mutua Terrassa, University of Barcelona, Barcelona, Spain; 8 Mental Health Services of Principado de Asturias (SESPA), Oviedo, Spain

**Keywords:** Bipolar disorder, Scientific community, Human behaviour

## Abstract

Bipolar disorder (BD) has been identified as a life-course illness with different clinical manifestations from an at-risk to a late stage, supporting the assumption that it would benefit from a staging model. In a previous study, we used a clustering approach to stratify 224 patients with a diagnosis of BD into five clusters based on clinical characteristics, functioning, cognition, general health, and health-related quality of life. This study was design to test the construct validity of our previously developed *k*-means clustering model and to confirm its longitudinal validity over a span of 3 years. Of the 224 patients included at baseline who were used to develop our model, 129 (57.6%) reached the 3-year follow-up. All life domains except mental health-related quality of life (QoL) showed significant worsening in stages (*p* < 0.001), suggesting construct validity. Furthermore, as patients progressed through stages, functional decline (*p* < 0.001) and more complex treatment patterns (*p* = 0.002) were observed. As expected, at 3 years, the majority of patients remained at the same stage (49.6%), or progressed (20.9%) or regressed (23.3%) one stage. Furthermore, 85% of patients who stayed euthymic during that period remained at the same stage or regressed to previous stages, supporting its longitudinal validity. For that reason, this study provides evidence of the construct and longitudinal validity of an empirically developed, comprehensive staging model for patients with BD. Thus, it may help clinicians and researchers to better understand the disorder and, at the same time, to design more accurate and personalized treatment plans.

## Introduction

Bipolar disorder (BD) is a life-course illness characterized by alternation of periods of euthymia with depressive, manic, and mixed episodes. Recently, it has been reconceptualized as a changing disorder with different clinical manifestations over the course of its development from an at-risk or latent stage to a late or end-stage, thus supporting the assumption that it would benefit from a staging model^1^. Clinical manifestations consistent with staging models include cognitive deterioration and functional decline^2,3^, changes in inflammatory and neuroanatomical biomarkers^3–9^, less response to treatment^10^, and worse self-reported quality of life (QoL)^11^ linked to disorder progression.

Although different staging models have been proposed from a theoretical perspective^12–16^ studies on BD with an empirical staging-development and longitudinal-data approach are scarce^17^. So far, only one study^18^ has tested the applicability of a theoretical clinical staging model for BD progressively developed by different authors^12–14^.

Recent research showed that BD could be fit to a mathematical model^19,20^. In a previous study, we developed a comprehensive, evidence-based *k*-means clustering model for BD that distinguishes five clusters ranging from the least severe (stage 1) to the most severe one (stage 5) based on clinical characteristics, physical health, cognition performance, real world functioning, and health-related QoL^21^. Now, with this study, we aim to use a different sample to test the construct validity of our model as well as its longitudinal validity, thereby providing proof of its validity as a staging model for use in patients with BD. We hypothesized that our model would behave properly with a different sample and that, at 3-year follow-up, the majority of the patients would remain at the same stage or would progress or regress one stage, while only a small proportion of patients would progress or regress two or more stages.

## Materials and methods

This is a prospective, 3-year follow-up, multicenter study conducted at four sites in Spain (Oviedo, Barcelona, and Valencia) with the aim to develop and validate an empirical staging model for using in patients with BD.

The baseline study was conducted between April 2012 and December 2014 (ref. PI11/02493), and the 3-year follow-up was conducted between April 2015 and July 2018 (ref. PI14/02037). The Clinical Research Ethics Committee of Hospital Universitario Central de Asturias in Oviedo approved the study protocol (refs. 36/12 and 142/15). Written informed consent was obtained from all participants prior to enrollment.

### Participants

Of the 224 patients enrolled at baseline, 129 (57.6%) completed the 3-year follow-up assessment. Inclusion criteria at baseline were: (1) outpatients with a SCID-I-confirmed diagnosis of BD according to DSM-IV-TR^22^ in treatment at any of the four participating sites; (2) age ≥ 18 years; and (3) written informed consent to participate in the study. Exclusion criteria consisted only of refusal to participate in the study.

### Assessments

Assessments were identical at baseline and at 3-year follow-up and included: (1) demographic and clinical information obtained from the clinical records of the patients (clinical course and specific characteristics of BD, psychiatric and physical comorbidities, officially recognized disability, and psychopharmacological treatments); (2) psychometric assessment: (2a) clinician-rated outcome measures (CROMs): Spanish versions of Hamilton Depression Rating Scale (HDRS)^23^, Hamilton Anxiety Rating Scale (HARS)^24^, Young Mania Rating Scale (YMRS)^25^, Clinical Global Impression (CGI)^26^, Oviedo Sleep Questionnaire (OSQ)^27^, Changes in Sexual Functioning Questionnaire (CSFQ)^28^, Scale for Cognitive Impairment in Psychiatry (SCIP)^29^, Global Assessment of Functioning (GAF)^30^, and Functioning Assessment Short Test (FAST)^31^; (2b) Patient-Reported Outcome Measures (PROMs): the Spanish version of MOS 36-item Short-Form Health-Survey (SF-36)^32^; (3) anthropometry [height, weight, waist circumference, and body mass index], vital signs (heart rate and blood pressure), and lab results [hematology (erythrocytes, hemoglobin, leukocytes, platelets), lipid profile (cholesterol, LDL cholesterol, HDL cholesterol, triglycerides), glucose, hepatic function (GPT, GOT, GGT, bilirubin), renal function (creatinine, BUN), hormones (PRL, TSH), and inflammatory and oxidative biomarkers (CRP, homocysteine)] were collected (for further detail, see Fuente-Tomas et al.^21^).

### Our staging model

The first step in the development of our staging model was to create a cluster-based method to classify patients with BD using a cross-sectional sample^21^. We made a dimensional reduction using *k*-means clustering. This technique aims to partition *n* observations into *k* clusters in which each observation belongs to the cluster with the nearest mean. Comparisons of between-group variables were then performed by Chi-square and univariate ANOVA followed by Tukey’s honestly significant difference post-hoc testing. Those variables in which statistically significant differences between groups were found were selected to be part of the model along with other variables added by expert criteria. We used all these variables, hereafter called profilers, to calculate a global severity formula.

Using the severity formula shown below, we obtain a global severity score for each patient which allows us to assign that patient to one of the five clusters of the staging model.

$${\mathrm {Severity}} = \frac{{10}}{{12}} \cdot \left( {{\mathrm {PD}}x{\mathrm {BD}} + {\mathrm {MetS}} + {\mathrm {ComPD}} + {\mathrm {SCIP}}_{{\mathrm {T}}_{r4}} +\; {\mathrm {IllnessN}} + {\mathrm {SFPF}} + {\mathrm {SFMH}} +\; {\mathrm {FAST}}_{\mathrm {T}} + {\mathrm {FAST}}_{\mathrm {leisure}} + {\mathrm {BMI}} + {\mathrm {HospN}} + {\mathrm {SuicAttN}}} \right)$$

The formula includes 12 profilers from the following five life domains: (1) Clinical characteristics of the BD: three profilers: Number of hospitalizations (HospN), Number of suicide attempts (SuicAttN), and Comorbid personality disorder (ComPD); (2) Physical health: three profilers: Body Mass Index (BMI), Metabolic Syndrome (MetS), and Number of comorbid physical illnesses (IllnessN); (3) Cognition: one profiler: Screen for Cognitive Impairment in Psychiatry score (SCIP_Tr4_); (4) real-world functioning: three profilers: permanently disabled due to BD (PD × BD), Functioning Assessment Short Test total score (FAST_T_), and Functioning Assessment Short Test leisure time subscale score (FAST_leisure_); and (5) Health-related QoL: two profilers: SF-36 Physical Functioning Scale score (SFPF), and SF-36 Mental Health Scale score (SF-MH). All profilers have the same weight and may take values between 0 and 1, so the severity score ranges from 0 to 10. Based on this score, we proposed the cut-off for delimiting the five clusters using the scores corresponding to the 5th, 25th, 50th, 75th, and 95th percentiles (1.70, 2.50, 4.50, and 6.10, and ≥6.11, respectively).

The second step, described in this paper, was to further validate our classification model as regards construct validity and longitudinal validity with the original sample at 3-year follow-up.

### Statistical analysis

Analyses were conducted using IBM SPSS Statistics for Windows, Version 22.0. The significance level was set at *p* < 0.05. We used a chi-squared test, paired *t*-test, and ANOVA with Tukey post-hoc test to identify associations between variables.

We tested for construct validity of our staging model by examining if: (1) all the profilers included in the model behave properly, that is, if patients get more severe scores on each profiler in late stages than in early stages and (2) our proposed external validators (GAF scores and pharmacological treatment patterns) also behave properly. We hypothesized that, in late stages, the global level of functioning would be more impaired and the prescribed pharmacological treatment more complex.

Concerning longitudinal validity, we analyzed the shift of patients throughout the model from baseline to 3-year follow-up. Here, we expected patients to move slightly forward or backward along the model with a very small percentage presenting greater changes (more than two stages). Furthermore, we expected a large proportion (more than 50%) of patients who stayed euthymic during the 3-year follow-up period to remain in the same stage.

## Results

On average, the mean follow-up time was 37.9 (SD = 2.1) months. At 3-year follow-up, 129 (57.6%) patients were reassessed.

### Demographic and clinical characteristics

Table 1 shows participant demographic and clinical characteristics, including the profilers of the model. Patients had a mean age of 50.3 (SD = 12.0), and the majority were female (65.2%) and Caucasian (96.2%). Diagnoses were as follows: 73% had BD I, 23 (17.4%) a comorbid personality disorder, and 9 (7%) a substance use disorder. Furthermore, 38 (32.2%) patients remained in a euthymic state throughout the follow-up period.

**Table 1:** Patient demographic and clinical characteristics.

Sample characteristics	Follow-up mean (SD) (*n* = 129)	Baseline mean (SD) (*n* = 129)
Mean age [mean (SD)]	50.3 (12.0)	46.9 (12.0)
Sex, females [*n* (%)]	86 (65.2)	86 (65.2)
Length of illness, years [mean (SD)]	23.6 (12.4)	20.5 (12.3)
Global assessment of functioning (GAF) [mean (SD)]	68.2 (14.0)	67.4 (15.5)
Suicide attempts^a^, yes [*n* (%)]	9 (6.8)	NA
Hospitalizations^a^, yes [*n* (%)]	23 (17.4)	NA
Manic episodes^a^, yes [*n* (%)]	25 (19.8)	NA
Depressive episodes^a^, yes [*n* (%)]	43 (33.9)	NA
Profilers (direct scores)		
Number of hospitalizations [mean (SD)]	1.2 (2.0)	2.3 (2.8)
Number of suicide attempts [mean (SD)]	1.0 (2.0)	0.9 (1.9)
Comorbid personality disorder, yes [*n* (%)]	23 (17.4)	24 (18.6)
Body mass index [mean (SD)]	24.5 (4.9)	28.7 (5.6)
Metabolic syndrome, yes [*n* (%)]	52 (39.4)	34 (27.6)
Number of comorbid physical illnesses [mean (SD)]	1.7 (1.6)	1.9 (1.2)
SCIP category, no cognitive impairment [*n* (%)]	51 (39.8)	34 (27.0)
Permanent disability due to bipolar disorder, yes [*n* (%)]	63 (47.8)	59 (45.7)
FAST total score [mean (SD)]	26.8 (15.8)	26.6 (16.8)
FAST leisure subscale score [mean (SD)]	2.2 (2.1)	3.0 (7.1)
SF-36 physical functioning scale score [mean (SD)]	−0.5 (0.7)	−0.5 (0.6)
SF-36 mental health scale score [mean (SD)]	−1.1 (2.1)	−1.1 (1.8)
Profilers (transformed score 0–1)		
Number of hospitalizations [mean (SD)]	0.19 (0.3)	0.37 (0.3)
Number of suicide attempts [mean (SD)]	0.18 (0.3)	0.15 (0.3)
Comorbid personality disorder, yes [*n* (%)]	0.18 (0.4)	0.18 (0.4)
Body mass index [mean (SD)]	0.15 (0.2)	0.32 (0.3)
Metabolic syndrome, yes [*n* (%)]	0.39 (0.5)	0.34 (0.5)
Number of comorbid physical illnesses [mean (SD)]	0.39 (0.3)	0.26 (0.3)
SCIP category, no cognitive impairment [*n* (%)]	0.47 (0.4)	0.40 (0.3)
Permanent disability due to bipolar disorder, yes [*n* (%)]	0.47 (0.5)	0.46 (0.5)
FAST total score [mean (SD)]	0.37 (0.2)	0.37 (0.2)
FAST leisure subscale score [mean (SD)]	0.33 (0.3)	0.40 (0.3)
SF-36 physical functioning scale score [mean (SD)]	0.53 (0.1)	0.55 (0.2)
SF-36 mental health scale score [mean (SD)]	0.62 (0.2)	0.55 (0.1)

^a^During the 3-year follow-up period.

The mean CGI-S score was 3.27 (SD = 1.4). Regarding psychopathology, 62 (47%) had a score consistent with bipolar depression according to the HDRS (≥7)^33^, 12 (9.1%) with a mixed episode (YMRS ≥ 7–20) and 4 (3%) with a manic episode according to the YMRS (>20). Concerning the cognitive assessment, 25 (19.4%) had mild, 27 (20.9%) moderate, and 26 (20.2%) severe impairment. On average, patients were receiving 3.2 (SD = 1.4) prescribed drugs. One hundred seventeen (90.7%) patients were taking one classic mood stabilizer, 29 (22.5%) were taking a combination of two, 78 (60.5%) at least one antipsychotic, 19 (14.7%) a combination of two, 51 (39.5%) antidepressants, and 65 (49.6%) benzodiazepines.

### Classification of the patients in the staging model

**Fig. 1:**
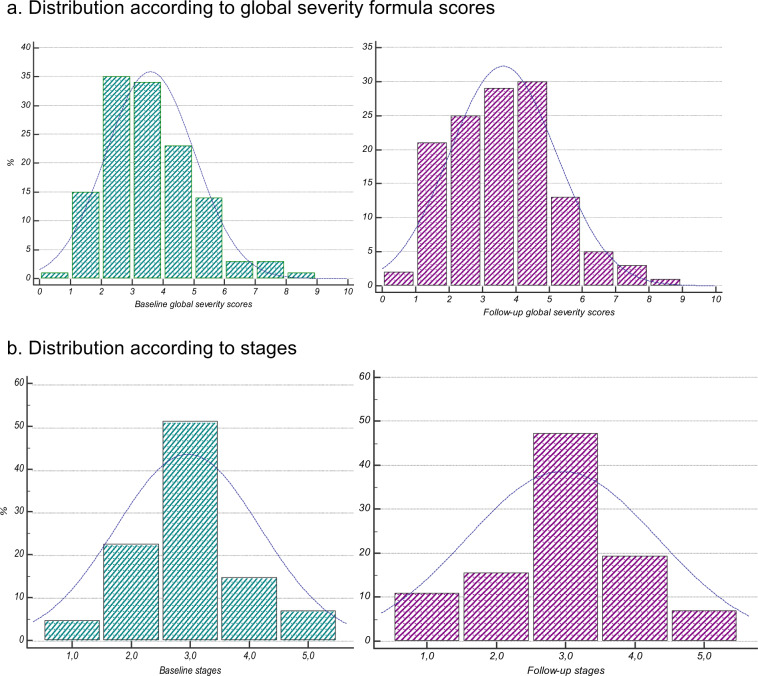
Distribution of patients according to staging model. **a** Distribution according to global severity formula scores and **b** distribution according to stages.

Of the 129 patients followed at 3 years, 14 (10.9%) were classified as stage 1, 20 (15.5%) as stage 2, 61 (47.3%) as stage 3, and 24 (18.6%) as stage 4, and 10 (7.8%) as stage 5. Their mean global severity score was 3.6 (SD = 1.6), with a minimum of 0.9 and a maximum of 8.4. At baseline, their mean global severity score was 3.6 (SD = 1.4), with a minimum value of 0.8 and maximum of 8.0 (see Fig. 1). The mean global severity score of patients who did not complete follow-up was 3.5 (SD = 1.3). We did not find significant differences in age, gender, bipolar type, age at onset, and total FAST score between followed and lost patients.

### Construct validity

As can be seen in Table 2, except for SF-36 mental health, all profilers became significantly worse as they progressed through the stages, thus providing proof of construct validity. Furthermore, evidence of construct validity was also provided by the external validators. Concerning GAF scores, significant worsening was seen as the stages progressed, ranging from 81.1 (SD = 11.9) in stage 1 to 49.5 (SD = 13.4) in stage 2 (see Table 3). Finally, regarding pharmacological treatment patterns, early stages (1 and 2) were associated with monotherapy or use of two-drug combinations, while late stages (4 and 5) were associated with combinations of four or more drugs (*p* = 0.002). Also, patients in late stages more frequently received antidepressants and benzodiazepines (see Table 3).

**Table 2:** Construct validity: values and distribution of profilers throughout the model.

Profilers	Stage 1 (*n* = 14)	Stage 2 (*n* = 20)	Stage 3 (*n* = 61)	Stage 4 (*n* = 24)	Stage 5 (*n* = 9)	Statistical test (df_b_, df_w_), *p*
Bipolar disorder						
Number of hospitalizations [mean (SD)]^a^	0.7 (0.3)	0.3 (0.6)	0.8 (1.1)	2.0 (1.9)	5.8 (4.2)	25.556^b^ (4, 124), <0.001
Number of suicide attempts [mean (SD)]^a^	0.1 (0.3)	0.3 (0.6)	0.5 (0.9)	1.8 (1.6)	5.6 (4.8)	26.236^b^ (4, 124), <0.001
Comorbid personality disorder, yes [*n* (%)]	0 (0.0)	0 (0.0)	27 (14.8)	8 (32.0)	6 (66.7)	23.850^c^, <0.001
Physical health						
Body mass index [mean (SD)]	21.2 (4.2)	21.6 (4.0)	25.3 (4.5)	26.0 (4.5)	28.5 (5.6)	7.012^b^ (4, 124), <0.001
Metabolic syndrome, yes [*n* (%)]	0 (0.0)	1 (5.0)	27 (44.3)	17 (68.0)	6 (66.7)	30.948^c^, <0.001
Number of comorbid physical illnesses [mean (SD)]^a^	0.3 (0.6)	0.8 (1.0)	1.9 (1.7)	2.5 (1.6)	2.4 (1.6)	7.827^b^ (4, 124), <0.001
Cognition						
SCIP category, no cognitive impairment [*n* (%)]	13 (92.9)	11 (55.0)	24 (40.0)	2 (8.0)	1 (11.1)	55.852^c^, <0.001
Real-world functioning						
Permanent disability due to BD, yes [*n* (%)]	0 (0.0)	4 (20.0)	30 (49.2)	18 (75.0)	9 (100.0)	35.107^c^, <0.001
FAST total score [mean (SD)	8.3 (6.7)	15.6 (10.2)	26.5 (11.4)	38.0 (11.7)	55.9 (9.2)	37.038^b^ (4, 122), <0.001
FAST leisure subscale score [mean (SD)]^a^	0.4 (0.6)	1.2 (1.8)	2.2 (1.9)	3.3 (2.0)	5.5 (0.9)	14.678^b^ (4, 122), <0.001
Health-related quality of life						
SF-36 physical functioning scale score [mean (SD)]^a^	0.3 (0,4)	0.2 (0.7)	−0.5 (1.0)	−0.8 (1.2)	−1.7 (1.1)	9.001^b^ (4, 122), <0.001
SF-36 mental health scale score [mean (SD)]	−0.7 (0.4)	−0.3 (0.6)	−0.6 (0.6)	−0.6 (0.7)	−0.5 (0.5)	1.112^b^ (4, 122), 0.354

*FAST* Functioning Assessment Short Test, *SCIP* Scale for Cognitive Impairment in Psychiatry, *SF-36* MOS 36-item Short-Form Health Survey.

^a^Variances among groups present statistically significant differences.

^b^ANOVA test.

^c^Chi-square test; (dfb, dfw) degree of freedom between subject and degree of freedom within subject.

**Table 3:** Construct validity, external validators: GAF scores and pharmacological treatment patterns throughout the model.

	Stage 1 (*n* = 14)	Stage 2 (*n* = 20)	Stage 3 (*n* = 61)	Stage 4 (*n* = 25)	Stage 5 (*n* = 9)	Statistical test (df_b_, df_w_), *p*
GAF scores [mean (SD)]	81.1 (11.9)	79.5 (9.2)	67.6 (11.4)	62.3 (10.8)	47.2 (12.0)	20.385^a^ (4,123), <0.001
Number of prescribed drugs [*n* (%)]						43.257^b^, 0.002
One drug	4 (30.8)	5 (26.3)	2 (3.7)	1 (4.5)	0 (0.0)	
Two drugs	5 (38.5)	8 (42.1)	14 (25.9)	3 (13.6)	0 (0.0)	
Three drugs	1 (7.7)	4 (21.1)	15 (27.8)	7 (31.8)	0 (0.0)	
Four drugs	2 (15.4)	1 (5.3)	12 (22.2)	5 (22.7)	5 (55.6)	
Five or more drugs	1 (7.7)	1 (5.3)	7 (13.0)	4 (18.2)	4 (44.4)	
Type of prescribed drugs [*n* (%)]						
Mood stabilizers						1.957^b^, 0.744
One drug	8 (66.7)	15 (83.3)	41 (80.4)	6 (28.6)	2 (28.6)	
Two drugs	4 (33.3)	3 (16.7)	10 (19.6)	9 (19.1)	7 (53.8)	
Antipsychotics	7 (50.0)	8 (40.0)	37 (60.7)	16 (66.7)	8 (80.8)	5.925^b^, 0.205
Antidepressants	3 (21.4)	6 (30.0)	31 (51.7)	11 (45.8)	9 (90.0)	13.960^b^, 0.007
Benzodiazepines	3 (21.4)	6 (31.6)	31 (50.8)	16 (66.7)	8 (80.0)	13.430^b^, 0.009

*GAF* global assessment of functioning.

^a^ANOVA test.

^b^Chi-square test; (dfb, dfw) degree of freedom between subject and degree of freedom within subject.

### Longitudinal validity

**Fig. 2:**
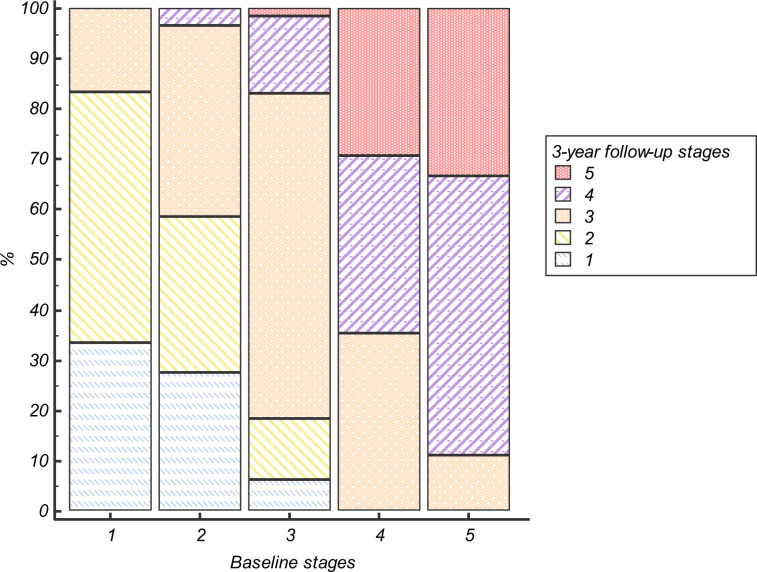
Shift throughout the model at 3 years of follow-up.

Figure 2 shows the shift of patients throughout the model at 3-year follow-up. Specifically, 50% of patients at stage 1 progressed to stage 2 and 16.7% to stage 3. Regarding stage 2, 27.6% of patients regressed one stage, while 37.9% progressed to stage 3 and only one (3.4%) advanced to stage 4. The majority of those at stage 3 remained at that stage (63.3%), while 18.2% regressed or progressed one stage. Regarding stage 4, 32% regressed to stage 3 and 26.3% progressed to stage 5. Finally, one-third of patients at stage 5 remained at that stage, while 55.6% regressed to stage 4 and 1 (11.1%) to stage 3.

When looking at the shifts in patients who stayed euthymic during the 3-year follow-up period, almost all remained at the same stage (55.3%) or regressed (23.7%) or advanced (15.8%) one stage. Two patients (5.3%) regressed two stages. In those patients who remained at the same stage or regressed to previous ones, there were statistically significant improvements in the clinical (*t* = 3.732, *p* = 0.001 and *t* = 5.090, *p* < 0.001, respectively), functioning (*t* = 2.626, *p* = 0.016 and *t* = 3.705, *p* = 0.004, respectively), and QoL dimensions (*t* = 8.000, *p* < 0.001 and *t* = 3.184, *p* = 0.010, respectively).

## Discussion

Our results demonstrate that our staging model has good construct and longitudinal validity, thus supporting its use in daily clinical practice. Regarding construct validity, with the exception of the mental health-related QoL profiler, all behave properly, showing significant worsening through the stages. Furthermore, the proposed external validators (GAF scores and pharmacological treatment pattern) also behave properly, that is, there is a functional decline across stages, and pharmacological treatment patterns are more complex at late stages than at early ones. Concerning longitudinal validity, at 3-year follow-up, the shift of patients throughout the model was as expected considering the short follow-up period, with half remaining at the same stage, 40% progressing or regressing one stage, and fewer than 10% progressing or regressing two.

Notwithstanding the fact that the course of BD is heterogeneous, there is evidence for clinical progression^34^, and accordingly, the five life domains of our staging model showed this progression. Regarding the clinical characteristics of BD, patients at late stages experienced more hospitalizations and suicide attempts and more frequently had a comorbid personality disorder. Consistent with these data, one study reported the same results between patients with first and multiple mood episodes^35^. However, two other studies^2,36^ did not find this clinical pattern among patients in different stages. This discrepancy may be due to the criteria used to classify patients into stages. In both of those studies, patients were assigned to the different stages based on functional impairment only, and not multiple domains of life. In addition, some of our profilers in this dimension were not used in those studies. Physical health, cognition, and functioning were the domains in which patients showed the most remarkable progressive worsening, thus identifying a score-dependent pattern. These findings comport with previous reports showing cognitive and functional decline along with the progression of BD^2,36,37^ and with theoretical models proposed by Kapczinski et al. (2009)^12^ and Cosci and Fava (2013)^38^. Concerning self-reported QoL, as the disorder progresses, physical QoL seems to worsen. In agreement with our results, a recent study by Tatay-Manteiga et al. (2019)^11^ showed that BD patients reported poorer QoL in late than early stages in physical, psychological, social, and environment domains. However, again, that study used a different criterion to classify patients based solely on FAST scores.

We have identified only one study that examined a staging model for BD from a longitudinal perspective^18^, although its aim was to find the patient characteristics that define their progression throughout the model. In our study, over the 3 years of follow-up, patients shifted across stages as expected, that is, in that very short period of time, only five patients had strong shifts (progressing or regressing two or more stages). Unfortunately, we did not find standardized patterns for transition over time in BD staging models to contrast with our results. Further proof of the longitudinal validity of our model is that most patients who were in a euthymic state remained at the same stage or had regressed to a previous one at follow-up. Although functional^39^ and cognitive^40^ impairments have been associated with subsyndromal depressive symptoms in cross-sectional studies, our longitudinal results demonstrate a statistically significant improvement in functioning and QoL dimensions in those patients who remained euthymic for 36 months and who remained at the same stage or regressed to a previous stage, thus calling into question the association reported in the literature.

All these findings support the construct and longitudinal validity of our model for patients with BD^21^ and provide further support for using this clinical staging model in clinical practice, taking into account the easy access to profiles in any clinical environment. We would like to highlight that our model is disorder‐specific, which contributes to the better understanding of BD. We did not include prodromal phases of the disorder because transdiagnostic staging models are probably better suited to the study of at-risk and prodromal phases, while disorder-specific models are more appropriate once it has been diagnosed^41^. However, the present results must be interpreted in light of one main limitation. Given that patients had to give signed informed consent prior to inclusion in the study, we were unable to include extremely severe/agitated patients in the model, consequently leading to underrepresentation of such patients in the model. Nevertheless, one of the main strengths of our study is its empirical approach and longitudinal prospective design. This is the first study to follow an entire range of adult patients representing different clinical stages of BD. Previous studies that validated the proposed models focused only on comparison of early vs. late stages, rather than on the full clinical course. Furthermore, our model considers BD a multidimensional disorder requiring five different life domains to classify patients, and the proposed severity classification formula is easy to implement in daily clinical practice.

In conclusion, this proposed staging model conforms to the conceptualization of BD as a progressive disorder that develops from mild to severe presentations. In this sense, it could help clinicians and researchers to better understand the disorder and, at the same time, to design more accurate and personalized treatment plans.
